# Advancing Pediatric Sarcomas through Radiomics: A Systematic Review and Prospective Assessment Using Radiomics Quality Score (RQS) and Methodological Radiomics Score (METRICS)

**DOI:** 10.3390/diagnostics14080832

**Published:** 2024-04-17

**Authors:** Gayane Aghakhanyan, Tommaso Filidei, Maria Febi, Salvatore C. Fanni, Andrea Marciano, Roberto Francischello, Francesca Pia Caputo, Lorenzo Tumminello, Dania Cioni, Emanuele Neri, Duccio Volterrani

**Affiliations:** 1 Department of Translational Research and of New Surgical and Medical Technology, University of Pisa, 56126 Pisa, Italy; 2 Department of Translational Research and of New Surgical and Medical Technology, Academic Radiology, University of Pisa, 56126 Pisa, Italydania.cioni@unipi.it (D.C.);; 3 Regional Center of Nuclear Medicine, University Hospital of Pisa, 56126 Pisa, Italy

**Keywords:** pediatric sarcoma, radiomics, diagnostic imaging, methodological rigor, Radiomics Quality Score (RQS), Methodological Radiomics Score (METRICS), imaging modalities, prognostic assessment, precision medicine

## Abstract

Pediatric sarcomas, rare malignancies of mesenchymal origin, pose diagnostic and therapeutic challenges. In this review, we explore the role of radiomics in reshaping our understanding of pediatric sarcomas, emphasizing methodological considerations and applications such as diagnostics and predictive modeling. A systematic review conducted up to November 2023 identified 72 papers on radiomics analysis in pediatric sarcoma from PubMed/MEDLINE, Web of Knowledge, and Scopus. Following inclusion and exclusion criteria, 10 reports were included in this review. The studies, predominantly retrospective, focus on Ewing sarcoma and osteosarcoma, utilizing diverse imaging modalities, including CT, MRI, PET/CT, and PET/MRI. Manual segmentation is common, with a median of 35 features extracted. Radiomics Quality Score (RQS) and Methodological Radiomics Score (METRICS) assessments reveal a consistent emphasis on non-radiomic features, validation criteria, and improved methodological rigor in recent publications. Diagnostic applications dominate, with innovative studies exploring prognostic and treatment response aspects. Challenges include feature heterogeneity and sample size variations. The evolving landscape underscores the need for standardized methodologies. Despite challenges, the diagnostic and predictive potential of radiomics in pediatric oncology is evident, paving the way for precision medicine advancements.

## 1. Introduction

Pediatric sarcomas, a complex array of rare malignancies originating from mesenchymal tissues, continue to pose significant challenges in diagnosis and treatment [1,2]. Among the common types are rhabdomyosarcoma, osteosarcoma, and Ewing sarcoma. Each subtype presents unique challenges in diagnosis and treatment, highlighting the need for tailored approaches to address the diverse spectrum of pediatric sarcomas [2]. These tumors often require multidisciplinary management involving surgery, chemotherapy, and/or radiation therapy to achieve optimal outcomes for affected patients. Further research into the molecular and genetic mechanisms underlying these tumors is essential for developing targeted therapies and improving survival rates in pediatric sarcoma patients [1]. Despite advancements in conventional imaging techniques, the intricacies of these tumors demand innovative solutions. Radiomics, a rapidly expanding field at the intersection of medical imaging and advanced data analytics, presents a promising avenue for unraveling the complexities of pediatric sarcomas [3,4]. Radiomics involves the extraction and analysis of a large number of quantitative features from medical images, including intensity, shape, texture, and spatial relationships, which are then subjected to sophisticated computational algorithms [3]. By discerning subtle patterns and associations within imaging data that may not be perceptible to the naked eye, radiomics holds the potential to provide valuable insights into the underlying biology, heterogeneity, and prognostic factors of pediatric sarcomas [4].

In this comprehensive review, we undertake an extensive appraisal of the current landscape of radiomics in the field of pediatric sarcomas, exploring its potential to reshape our understanding of these malignancies and refine clinical management strategies. The quality of radiomics studies represents a pivotal landmark for advancing radiomic research and future clinical applications. To this end, the Radiomics Quality Score (RQS), proposed by Lambin et al. as a robust tool for evaluating the quality of radiomics studies, was applied to evaluate the reliability and reproducibility of radiomic findings [5]. Anchored in 16 items related to the key steps of the radiomic workflow, the RQS offers a structured framework to critically assess methodological rigor, ensuring a comprehensive evaluation of the reliability and reproducibility of radiomic findings.

Acknowledging the dynamic nature and current development in the field of radiomics, we further extend our exploration to include the recently released quality assessment tool, the Methodological Radiomics Score (METRICS) [6]. Developed by an international consortium of domain experts, the METRICS employs a flexible format covering all methodological variations, providing a well-constructed framework for assessing the quality of radiomic research papers. EuSoMII, the European Society of Medical Imaging Informatics (https://www.eusomii.org/; accessed on 1 March 2024), has endorsed METRICS as a valuable tool for evaluating the methodological quality of radiomics studies. We aim to incorporate both the RQS and METRICS to offer a comprehensive view of radiomics studies in the field of pediatric sarcomas, providing insights into the methodological quality and rigor of these studies.

Our manuscript aims to contribute to the ongoing discourse surrounding the integration of radiomics into routine clinical practice for pediatric sarcomas. By incorporating both the RQS and METRICS, we aspire to provide a holistic evaluation of radiomics studies, specifically within the context of pediatric sarcomas, offering a nuanced perspective on their quality and potential impact in guiding therapeutic decisions for pediatric sarcoma patients. Our ultimate objective is to foster a deeper understanding of the field, thereby facilitating the translation of radiomics from theoretical promise to tangible improvements in pediatric oncology outcomes.

## 2. Materials and Methods

### 2.1. Systematic Search Strategy

A systematic review was conducted to identify original research papers pertaining to radiomics analysis in pediatric sarcoma, published up to November 2023. The search encompassed the PubMed/MEDLINE (*n* = 26), Web of Knowledge (*n* = 17), and Scopus (*n* = 29) databases. The search strategy employed the following terms: (histogram) OR (texture) OR (textural) OR (radiomics)) AND (sarcoma [MeSH Terms]) AND (pediatric). This review was performed in accordance with the PRISMA (Preferred Reporting Items for Systematic Reviews and Meta-Analyses) guidelines.

### 2.2. Eligibility Criteria and Study Selection

The selection of studies for inclusion in this review aimed to comprehensively evaluate the application of radiomics in pediatric sarcomas. The following eligibility criteria were predefined to ensure the relevance and quality of the included literature:

1. Publication Type: Peer-reviewed journal articles reporting original research studies were included. Conference abstracts, editorials, letters, and reviews were excluded.
2. Study Design: Studies employing radiomics methodology for the analysis of medical imaging data in pediatric patients with sarcomas were eligible for inclusion. Both retrospective and prospective studies were considered.
3. Population: Studies involving pediatric patients (aged ≤ 18 years) diagnosed with sarcomas of various subtypes, including but not limited to osteosarcoma, Ewing sarcoma, rhabdomyosarcoma, and liposarcoma, were included.
4. Outcome Measures: Studies reporting on the application of radiomic features extracted from medical imaging modalities such as MRI, CT, or PET/CT and PET/MRI for the diagnosis, prognosis, treatment response assessment, or predictive modeling of pediatric sarcomas were included.
5. Language: Studies published in English were considered for inclusion in this review.

### 2.3. Data Extraction

Data extraction involved collecting various details from the included studies focusing on pediatric sarcomas. These details encompassed authors’ names, the year of publication, and the title of the publication. The full manuscripts were assessed to retrieve additional critical information. This included discerning the study type as either retrospective or prospective and determining the total number of patients included. Moreover, specific details regarding the type of sarcomas (e.g., Ewing sarcoma, osteosarcoma, rhabdomyosarcoma, etc.), imaging modalities employed (such as CT, MRI and/or PET/CT, PET/MRI), single or multiple sequence modalities applied for feature extraction, the software utilized for segmentation and imaging analysis, the segmentation method (manual, semiautomated and fully automated), and the total number of radiomic features extracted were documented. 

To enhance clarity, the studies were further categorized based on their primary investigative objectives. These objectives encompassed diagnostic studies, which included radiomics analyses for differential diagnosis and the prediction of tumor histopathological differentiation. Additionally, studies focusing on prognostic factors, aiming to predict early recurrence and survival outcomes, as well as those investigating treatment responses, were grouped for comprehensive analysis.

### 2.4. Analysis of the Quality Based on Radiomics Quality Score (RQS)

The methodological quality of the studies included in our analysis underwent thorough assessment by a panel of three expert reviewers: G.A., a nuclear medicine specialist with 7 years of experience, and M.F. and T.F., both accomplished radiologists with 3 and 1 years of experience, respectively. Employing the RQS framework, as proposed by Lambin et al. [5], our evaluation aimed to scrutinize the methodological robustness of each study.

The RQS serves as a comprehensive tool designed to gauge the methodological strength of radiomics studies, as extensively detailed in previous research [4,7,8,9]. Comprising 16 distinct items, it encompasses critical facets of study design, image acquisition and preprocessing, feature extraction, feature selection, model building, and validation methods. Each item within the RQS is evaluated against predefined criteria, with higher scores reflecting superior methodological quality. The total RQS (ranging from −8 to + 36) and the percentage of the total score (0–100%) were recorded from all three readers. In instances where discrepancies arose among the three reviewers, consensus was reached through majority decision-making, ensuring a rigorous and consistent evaluation process.

### 2.5. Analysis of the Methodological Radiomics Score (METRICS)

The METRICS is a quality scoring tool for evaluating the methodological rigor of radiomics studies (https://metricsscore.github.io/metrics/METRICS.html; accessed on 1 March 2024) [6]. It consists of 30 items within 9 categories that assess various aspects of study methodology, including study design, image acquisition and preprocessing, feature extraction, feature selection, model building, and validation techniques. Each item is scored based on predefined criteria, with higher scores indicating better methodological quality. The 30 items in METRICS offer a comprehensive assessment of the key components necessary for robust radiomics research. The total METRICS, expressed as a percentage, is calculated as a representation of the overall methodological quality of a radiomics study. The METRICS percentage value ranges from 0 to 100 and is derived from the sum of individual item scores divided by the maximum possible score. This percentage score provides a quantitative measure of the methodological quality, allowing for comparative analysis across different studies.

To facilitate interpretation, the METRICS percentage is categorized into five arbitrary categories representing gradually increasing quality:

- Very Low Quality (0 ≤ score < 20%): Radiomics studies falling within this category exhibit very low methodological quality, indicating significant deficiencies in study methodology.
- Low Quality (20 ≤ score < 40%): Studies categorized as low quality demonstrate an improvement over very low quality but still exhibit notable shortcomings in methodological rigor.
- Moderate Quality (40 ≤ score < 60%): Studies in the moderate quality category indicate a satisfactory level of methodological rigor, with noticeable improvements compared to low-quality studies.
- Good Quality (60 ≤ score < 80%): Studies classified as good quality demonstrate a significantly improved level of methodological rigor, with strong adherence to established guidelines and standards.
- Excellent Quality (80 ≤ score ≤ 100%): Studies achieving excellent quality represent the highest level of methodological rigor, exhibiting exceptional adherence to best practices and standards in radiomics research.

### 2.6. Statistical Analysis

The level of inter-rater agreement of RQS total percentages among three independent raters was assessed using Fleiss’ Kappa statistic, suitable for multiple raters [10]. It is commonly used when dealing with more than two raters and provides a measure of agreement that considers chance agreement among all raters. The inter-rater agreement for the scoring categories of the METRICS was assessed using Cohen’s Kappa statistic, suitable for two raters. Cohen’s Kappa provides a measure of agreement that adjusts for the possibility of chance agreement between raters, thus providing a more robust assessment of inter-rater reliability. The interpretation of both Fleiss’ Kappa and Cohen’s Kappa values is as follows: values less than 0 were categorized as no agreement, 0.01–0.20 as slight agreement, 0.21–0.40 as fair agreement, 0.41–0.60 as moderate agreement, 0.61–0.80 as substantial agreement, and 0.81–1.00 as almost perfect agreement.

Additionally, the total METRICS was evaluated using the intraclass correlation coefficient (ICC) with 95% confidence intervals (CIs). The ICC was calculated based on an absolute agreement with a 2-way mixed-effect model, which accounts for both systematic and random variations among readers. The interpretation of ICC values followed established guidelines: poor agreement for ICC < 0.50, moderate agreement for ICC = 0.50–0.75, good agreement for ICC = 0.75–0.90, and excellent agreement for ICC > 0.90.

## 3. Results

### 3.1. Study Selection

**Figure 1:**
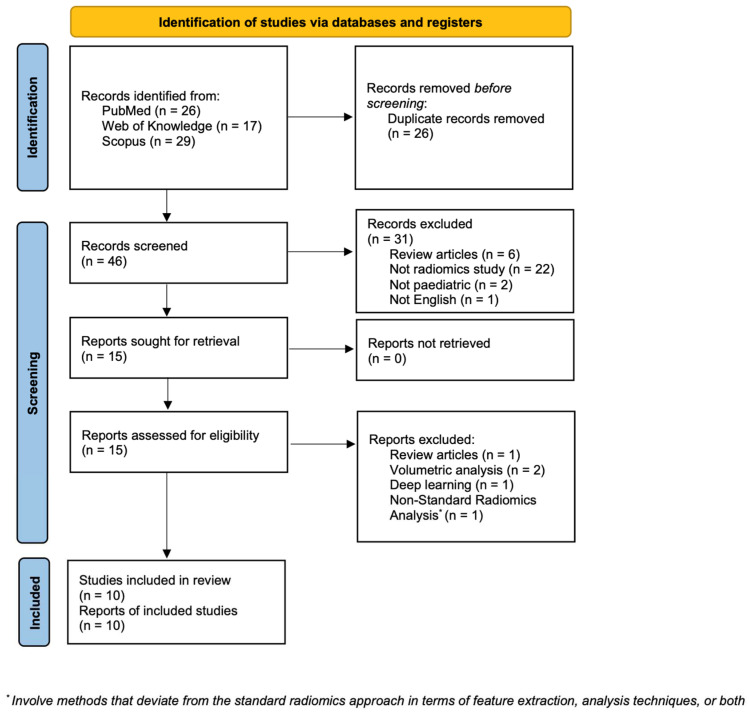
PRISMA flow diagram.

Initially, a total of 72 papers were identified using the specified search terms. Subsequently, 26 duplicate articles were excluded, resulting in 46 unique articles for further evaluation. Among these, 31 articles were excluded based on predefined criteria: 6 were review articles, 22 were studies that were unrelated to radiomics, 2 were not focused on pediatric populations, 1 was not written in English, and 2 were centered on volumetric diffusion-weighted imaging (DWI) analysis, with an additional article focused on deep learning and another one involving methods that deviate from the standard radiomics approach in terms of feature extraction and analysis techniques. Finally, following the application of inclusion and exclusion criteria, a total of 10 reports were included in the review process (refer to Figure 1 for the selection process flowchart) [11,12,13,14,15,16,17,18,19,20].

### 3.2. Characteristics of Included Studies

The characteristics of the included studies are summarized in Table 1. These studies were published between 2017 and 2023. Among the included publications, the year 2017 had the fewest publications (*n* = 1), while 2022 had the greatest number of studies (*n* = 4). All of the studies were retrospective in nature and predominantly focused on Ewing sarcoma (EWS) and osteosarcoma (OST). The imaging modalities utilized in the included studies were CT (three studies), MRI (four studies), PET/CT (two studies), and PET/MRI (two studies). Two studies used multiple sequence modalities for feature extraction. 

In the reviewed studies, the extraction of radiomic features was predominantly conducted using widely employed software tools such as LifeX, ITK-SNAP, and 3D-Slicer for the robust analysis of texture features. The majority of the studies included region-of-interest (ROI) segmentation methods, with manual segmentation being the most commonly employed method (*n* = 7), followed by two studies that used semiautomated methods and one study that utilized a fully automated method.

The studies included in this review covered a total of 628 participants. The sample size across the studies ranged from 15 to 176 participants, with a median of 62 participants. In terms of feature extraction, a median of 35 features were extracted across the studies. The majority of the articles reported a limited number of features, with only two articles extracting 851 [20] and 342 [14] features. Most of the studies focused on feature extraction from primary tumors, while one study analyzed lung metastases [15]. Based on the primary investigative objectives, five papers were focused on diagnostic purposes, three on prognostic assessment, two on treatment response evaluation, and one additional paper focused on other aspects such as reduced injected tracer activities [11].

### 3.3. Quality Assessment Using RQS

The inter-rater agreement among the three independent raters for the RQS total percentage using Fleiss’ Kappa yielded a substantial agreement (Kappa = 0.478) among the raters. The high z-value (7.71) and low *p*-value (*p* < 0.05) indicated significant agreement beyond chance. 

**Figure 2:**
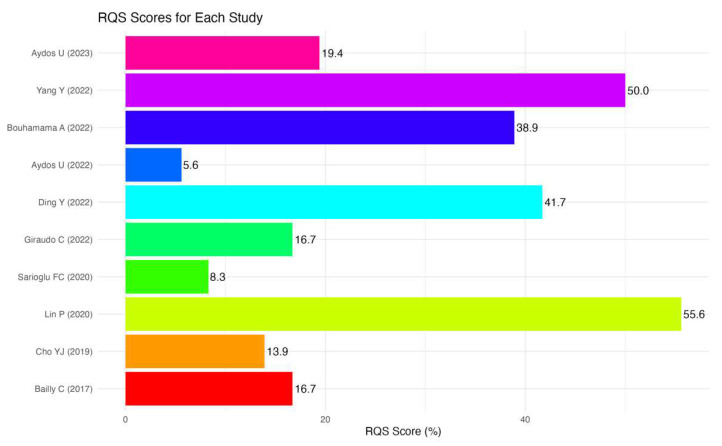
Radiomics Quality Score (RQS) assessment. The horizontal bar chart presents the distribution of RQS total % across different studies. Each colored bar corresponds to a specific study, with the length of the bar indicating the RQS. The chart provides a visual comparison of RQSs, aiding in the assessment of variability and central tendency among the studies. Study names are listed on the *y*-axis, and RQSs are indicated on the *x*-axis [11,12,13,14,15,16,17,18,19,20].

**Figure 3:**
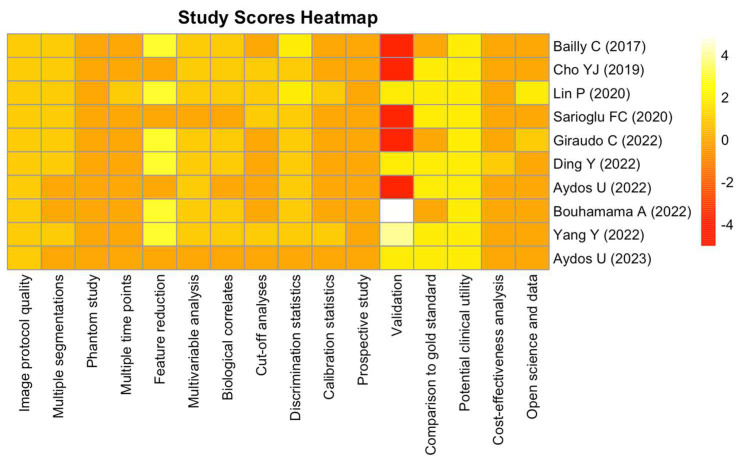
The heatmap illustrates the RQS criteria for different studies. Each row represents a study, and each column corresponds to a specific criterion. The color intensity indicates the score for each criterion, ranging from low (red) to high (yellow/white). Criteria names are provided on the *x*-axis, while the study names are on the right side of the heatmap [11,12,13,14,15,16,17,18,19,20].

The incorporated studies demonstrated a median RQS of 6.5 points, equivalent to 18.1% when expressed as a percentage (Figure 2). The scores ranged from 3 to 20, representing a spectrum from 8.3% to 55.6%, respectively. Figure 3 provides a detailed breakdown of these scores. Notably, four criteria, namely discrimination statistics, the potential clinical utility, retrospective design, and a well-documented image protocol, were consistently scored higher across the studies. Conversely, three criteria, namely feature reduction or adjustment for multiple testing, validation and detection, and the discussion of biological correlates, were less frequently met.

A trend observed across some studies involved the incorporation of non-radiomic features into the analysis (criterion 6), often compared to established gold standards (criterion 13). Notably, studies conducted in 2022 and 2023 placed particular emphasis on the validation criterion (criterion 12). 

### 3.4. Methodological Rigor Assessment Using METRICS

The assessment of inter-rater agreement in this study utilized Cohen’s kappa coefficient for the determination of agreement on quality categories within the METRICS framework. The results revealed a robust level of agreement between the two readers, with a statistically significant Cohen’s kappa coefficient of 0.833 (z = 3.69, *p*-value = 0.000228). This finding underscores a high degree of consensus in the qualitative categorization of the reviewed papers. Table 2 provides a comparison of the total METRICS and quality categories assigned by two readers (Reader 1 and Reader 2) for each study.

**Figure 4:**
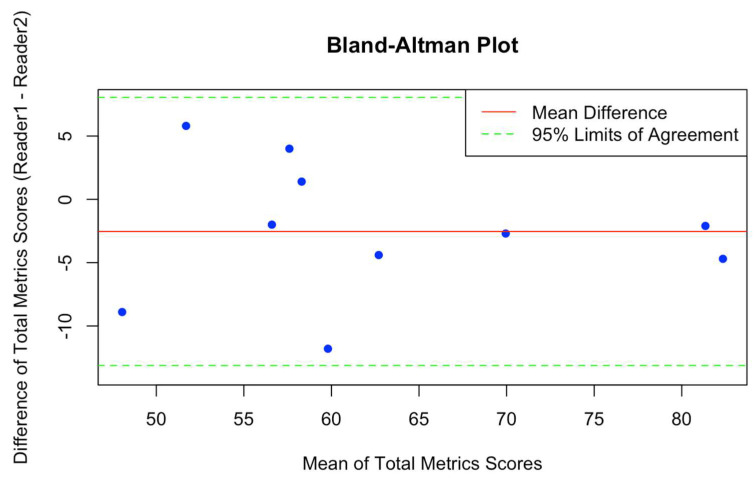
The Bland–Altman plot illustrates the agreement between two readers (Reader 1 and Reader 2) for assessing the total METRICS. Each data point represents the difference in scores between the two readers (Reader 1 and Reader 2) plotted against the mean of the scores from both readers. The red line represents the mean difference between the scores, while the green dashed lines indicate the 95% limits of agreement (mean difference ± 1.96 * standard deviation of differences). Points falling within the limits of agreement suggest good agreement between the readers, while points outside the limits indicate potential discrepancies.

In addition, the inter-rater agreement for the total METRICS was evaluated through the application of the ICC. The outcome of this analysis indicated a noteworthy level of concordance between the two raters, as evidenced by a substantial ICC value of 0.886. The statistical significance of this coefficient was established with a very low *p*-value (*p* = 7.12 × 10^−5^), affirming the robustness of the observed agreement. The 95% confidence interval for ICC values, ranging from 0.627 to 0.97, provides additional insight into the precision of the ICC estimate. This interval suggests a high level of confidence in the reliability and consistency of the total METRICS assessments made by the two readers. The Bland–Altman plot (Figure 4) illustrates the agreement between two readers (Reader 1 and Reader 2) for assessing the total METRICSs.

The analysis of quality categories within the METRICS framework across the reviewed publications reveals the following patterns: The majority of the publications are predominantly classified as “moderate”, representing the most prevalent quality category assigned by both readers. By contrast, fewer papers fall into the “excellent” category [14,20], indicating a lower frequency of publications achieving the highest quality assessment. Notably, an interesting temporal trend emerges when considering the quality categories over the years. The “excellent” category appears to have witnessed an increase in representation in more recent publications, suggesting an improvement in the methodological and reporting standards of studies within the 2022–2023 timeframe.

Examining the median total METRICS provides additional insights. The publications show a median score of 59.3, with the range spanning from 43.6 to 80.3. The lowest score of 43.6 suggests the presence of publications with moderate methodological quality [13], while the highest score of 80.3 indicates the existence of studies attaining an excellent quality rating [20]. This wide range underscores the diversity in the methodological rigor of the included papers, showcasing variances in the overall quality of radiomics research within the assessed dataset.

## 4. Discussion

The primary objective of this systematic review is to evaluate the evolving landscape of radiomics in pediatric sarcoma patients, addressing methodological considerations and exploring key applications such as diagnostics and predictive modeling. The rigorous selection process led to the inclusion of 10 studies, offering a snapshot of the current landscape of pediatric sarcoma radiomics research. The included studies span a timeframe from 2017 to 2023, showcasing a growing interest in the application of radiomics in pediatric oncology over the years. This temporal evolution is highlighted by a notable surge in publications, particularly evident in 2022, which emerged as the year with the highest number of studies included in this review. The studies predominantly adopt a retrospective design and focus primarily on Ewing sarcoma and osteosarcoma, aligning with the prevalent malignancies in the pediatric population [21]. Notably, a variety of imaging modalities are employed, ranging from CT and MRI to PET/CT and PET/MRI, reflecting the diverse technological landscape in pediatric oncologic imaging. The integration of multiple sequence modalities for feature extraction in a small subset of studies [19,20] further exemplifies the nuanced and multifaceted approach employed by researchers in characterizing pediatric tumors.

### 4.1. Methodological Transparency

A pivotal aspect of the included studies lies in their commitment to methodological transparency. Manual segmentation emerges as the predominant method for region-of-interest (ROI) delineation. The overall patient dataset, encompassing 628 participants, exhibits a wide range of sample sizes, emphasizing the variability in study populations across the pediatric sarcoma radiomic landscape. Feature extraction, a cornerstone in radiomics research, reveals a median of 35 features extracted across the studies. While the majority of the articles adhere to a focused selection of features, a few studies stand out, extracting a notably higher number of features. This diversity in feature selection underscores the methodological heterogeneity within the field, thus necessitating a comprehensive interpretation of results.

Quality assessment using the RQS unfolds a detailed picture of the strengths and limitations of the included studies [22]. The inter-rater agreement among independent raters attests to the consistent application of the RQS tool, emphasizing its utility in promoting standardized quality evaluation. The examination of RQSs reveals specific criteria in which studies demonstrate excellence and areas that require improvement. The observed trends, such as the consistent emphasis on non-radiomic features and the growing attention to validation criteria in more recent years, highlight the evolving landscape of pediatric oncologic radiomics research.

The evaluation of methodological rigor through the METRICS framework not only reaffirms the reliability of the included studies but also demonstrates a high level of agreement between readers, as indicated by Cohen’s kappa coefficient and the ICC. This robust and consistent qualitative and quantitative evaluation is further exemplified in the detailed comparison of total METRICSs and quality categories assigned by the two readers, enhancing the overall reliability of the study assessments. In comparison to the RQS, the METRICS framework appears more reliable, as evidenced by the demonstrated high level of inter-rater agreement [23].

### 4.2. Primary Investigative Objectives 

This review encompasses studies with a primary focus on diagnostic applications, showcasing the pivotal role of radiomics in characterizing pediatric tumors across diverse imaging modalities such as CT, MRI, PET/CT, and PET/MRI. Notably, Sarioglu et al. utilized MRI texture analysis (TA) to distinguish pediatric craniofacial rhabdomyosarcoma from infantile hemangioma (IH). Their findings highlighted the potential of TA, particularly the gray-level zone length matrix parameters, as predictors for rhabdomyosarcoma [19]. Similarly, Ding et al. conducted a radiomics analysis to differentiate Kaposiform hemangioendothelioma (KHE) and fibro-adipose vascular anomaly (FAVA) in extremities [16]. Their MRI-based radiomic model demonstrated significant differentiating capacity. Giraudo et al. presented a PET/MR-based application of radiomics for pediatric soft tissue sarcoma, revealing the discriminative power of specific radiomic features in classifying tumors of different grades and histotypes [17]. Radiomics was applied not only for primary tumors but also for metastases, as demonstrated by Cho et al., who focused on CT imaging for the differentiation of pulmonary metastases in children with osteosarcoma. They introduced a 3D radiomic technique with superior diagnostic performance compared to conventional measurements [15].

Extending beyond diagnostic applications, Yang et al. incorporated prognostic assessment in their study. Their multimodality imaging-derived models, utilizing computer-aided diagnostic (CAD) methods, demonstrated robust predictive performance for identifying well-differentiated liposarcoma (WDLPS) and lipoma [20]. They employed both handcrafted radiomics analysis and deep learning techniques, emphasizing the potential of advanced methodologies in prognostic evaluation. Further addressing prognostic aspects, Bailly et al. evaluated the FDG-PET-derived radiomic metrics for a homogeneous pediatric Ewing sarcoma and osteosarcoma population [13]. Although no prognostic value was found for Ewing sarcoma, a shape feature (elongation) in osteosarcoma proved significant for both progression-free and overall survival.

Predicting treatment response through radiomics has emerged as a crucial aspect of pediatric oncology, offering insights into the effectiveness of therapeutic interventions. In this context, Lin P et al. developed a delta-radiomic signature-based nomogram for evaluating preoperative chemotherapeutic response in high-grade osteosarcoma, which outperforms single-CT-based radiomic signatures [18]. Similarly, Bouhamama et al. focused on predicting neoadjuvant chemotherapy response using MRI-based radiomics, achieving high predictive accuracy [14]. The exploration of treatment response in these studies contributes to both prognostication and the ongoing refinement of radiomic applications in pediatric oncology.

Collectively, the studies underscore the diagnostic and predictive potential of radiomics in pediatric sarcoma research. The shared emphasis on discrimination statistics across studies enhances diagnostic precision. Noteworthy contributions from Yang et al., Bailly et al., Lin P et al., and Bouhamama et al. highlight significant associations in prognostic evaluation and innovative approaches for treatment response assessment [13,14,18,20]. The varied imaging modalities and advanced techniques employed underscore radiomics’ evolving role in comprehensively understanding and managing pediatric tumors.

### 4.3. Current Landscape, Challenges, and Opportunities

The contemporary landscape of pediatric oncologic radiomics research is characterized by a pressing need for standardized methodologies and increased methodological transparency. As the field continues to expand, there is a growing recognition of the importance of incorporating non-radiomic features into the analysis, aligning with the broader trend of integrating multidimensional data for a holistic understanding of pediatric tumors. The observed temporal trend toward emphasizing validation criteria in more recent studies underscores the field’s commitment to enhancing the reliability and reproducibility of radiomic findings. The continuous evolution of imaging modalities and technological advancements necessitates an adaptive approach to feature extraction, ensuring that radiomics research remains at the forefront of precision medicine in pediatric oncology.

While the included studies showcase commendable efforts, challenges such as the heterogeneity in feature selection, sample sizes, and the need for standardized reporting persist. These challenges present opportunities for future research to focus on establishing consensus guidelines, fostering collaboration, and refining methodological frameworks.

In conclusion, this systematic review provides a comprehensive assessment of the evolving role of radiomics in pediatric oncology. By examining the methodological landscape and exploring key applications such as diagnostics and predictive modeling, we have shed light on the potential of radiomics to enhance clinical decision-making and patient care in pediatric sarcoma patients. The observed trends underscore the growing interest and utility of radiomics in characterizing tumor heterogeneity and predicting treatment response. However, while the promising findings showcased in the reviewed studies suggest a promising future for radiomics in pediatric oncology, challenges such as standardization, validation, and integration into clinical practice remain. Moving forward, concerted efforts are warranted to address these challenges and fully leverage the clinical potential of radiomics as a valuable tool for personalized medicine in pediatric sarcomas.

## Figures and Tables

**Table 1:** Study characteristics.

First Author	Year	Title	Study Design ^#^	Sample Size	Tumor Type	Analysis Strategy *	Software	Radiomic Feature	Feature Type	Sequences	Imaging Modality	Segmentation **	Primary Investigative Objectives
Bailly et al. [13]	2017	Prognostic Value of FDG-PET Indices for the Assessment of Histological Response to Neoadjuvant Chemotherapy and Outcome in Pediatric Patients with Ewing Sarcoma and Osteosarcoma	R	62	Ewing sarcoma, osteosarcoma	U; M	PLANE-T1Onco-Solution; https://www.dosisoft.com/products/planet-onco/	9	Heterogeneity (GLCM, GLRLM, and GLSZM), Shape features	single	[18F]FDG PET/CT	3D, A	prognostic
Cho et al. [15]	2019	Computerized Texture Analysis of Pulmonary Nodules in Pediatric Patients with Osteosarcoma: Differentiation of Pulmonary Metastases from Non-Metastatic Nodules	R	16	Osteosarcoma	U	MISSTA; in-house software program	12	first-order, second-order texture, and morphologic features	single	CT	2D, M	diagnostic
Lin et al. [18]	2020	A Delta-Radiomics Model for Preoperative Evaluation of Neoadjuvant Chemotherapy Response in High-Grade Osteosarcoma	R	191	Osteosarcoma	M	ITK-SNAP; http://www.itksnap.org/pmwiki/pmwiki.php	68	Intensity, texture, and wavelet features	single	CT	3D, M	treatment response
Sarioglu et al. [19]	2020	MRI-Based Texture Analysis for Differentiating Pediatric Craniofacial Rhabdomyosarcoma from Infantile Hemangioma	R	15	Craniofacial rhabdomyosarcoma	U	LifeX; https://www.lifexsoft.org	38	texture features	multiple	MRI	2D, M	diagnostic
Ding et al. [16]	2020	MRI-Based Radiomics in Distinguishing Kaposiform Hemangioendothelioma (KHE) and Fibro-Adipose Vascular Anomaly (FAVA) in Extremities: A Preliminary Retrospective Study	R	30	Kaposiform hemangioendothelioma	M	3D Slicer; https://www.slicer.org	107	Shape and first- and second-order features	single	MRI	3D, M	diagnostic
Giraudo et al. [17]	2022	Radiomic Features as Biomarkers of Soft Tissue Paediatric Sarcomas: preliminary results of a PET/MR study	R	18	Soft tissue sarcomas (mainly rhabdomyosarcomas)	U	3D Slicer; https://www.slicer.org	33	First- and second-order features	single	[18F]FDG PET/MRI	3D, M	diagnostic
Aydos et al. [12]	2022	Prognostic Value of Fluorodeoxyglucose Positron Emission Tomography-Derived Metabolic Parameters and Textural Features in Pediatric Sarcoma	R	43	Osteosarcoma, Ewing sarcoma, rhabdomyosarcoma	U; M	LifeX; https://www.lifexsoft.org	15	Histogram, GLRLM, GLCM, NGLDM, DLZLM	single	[18F]FDG PET/CT	3D, SA	prognostic
Bouhamama et al. [14]	2022	Prediction of Histologic Neoadjuvant Chemotherapy Response in Osteosarcoma Using Pretherapeutic MRI Radiomics	R	176	Osteosarcoma	ML; DL	ITK-SNAP; http://www.itksnap.org/pmwiki/pmwiki.php	342	Size, shape, and texture features	single	MRI	3D, M	treatment response
Yang et al. [20]	2022	Novel Computer-Aided Diagnostic Models on Multimodality Medical Images to Differentiate Well-Differentiated Liposarcomas from Lipomas Approached by Deep Learning Methods	R	58	Well-differentiated liposarcoma	U; M; ML; DL	ITK-SNAP http://www.itksnap.org/pmwiki/pmwiki.php	851	First-order; shape and second-order features	multiple	CT; MRI	3D, M	diagnostic, prognostic
Aydos et al. [11]	2023	Quantitative and Visual Analyses of the Effect of Activity Reduction on Image Metrics and Quality in 18F-FDG PET/MRI in Pediatric Oncology	R	19	Sarcoma	U	LifeX; https://www.lifexsoft.org	32	Histogram, GLRLM, GLCM, NGLDM, DLZLM	single	[18F]FDG PET/CT	3D, SA	other (reduced injected tracer activities)

Abbreviations: [18F]FDG PET/CT: fluorodeoxyglucose positron emission tomography/computed tomography; CT: computed tomography; DLZLM: dependence size zone matrix; GLCM: gray-level co-occurrence matrix; GLRLM: gray-level run-length matrix; GLSZM: gray-level size zone matrix; MISSTA, medical imaging solution for segmentation and texture analysis; MRI: magnetic resonance imaging; NGLDM: neighboring gray-level dependence matrix. ^#^ Study design: R = retrospective; P = prospective. * Analysis strategy: U = univariate feature analysis, M = multivariate prediction models, ML = machine learning, DL = deep learning. ** ROI segmentation method: M = manual, SA = semiautomatic, A = automatic.

**Table 2:** A comparison of the total METRICS and quality categories assigned by two readers (Reader 1 and Reader 2) for each author’s study.

	Reader 1		Reader 2	
First Author	Total METRICS	Quality Category	Total METRICS	Quality Category
Bailly C (2017) [13]	43.6	Moderate	52.5	Moderate
Cho YJ (2019) [15]	59.6	Moderate	55.6	Moderate
Lin P (2020) [18]	68.6	Good	71.3	Good
Sarioglu FC (2020) [19]	54.6	Moderate	48.8	Moderate
Giraudo C (2022) [17]	53.9	Moderate	65.7	Good
Ding Y (2022) [16]	60.5	Good	64.9	Good
Aydos U (2022) [12]	55.6	Moderate	57.6	Moderate
Bouhamama A (2022) [14]	80.3	Excellent	82.4	Excellent
Yang Y (2022) [20]	80.0	Excellent	84.7	Excellent
Aydos U (2023) [11]	59.0	Moderate	57.6	Moderate

## Data Availability

The data supporting the conclusions of this article will be made available by the authors upon request.

## References

[1] Sandler G., Yokoi A., Hayes-Jordan A. (2019). An Update in the Management of Pediatric Sarcoma. Curr. Opin. Pediatr..

[2] Williams R.F., Fernandez-Pineda I., Gosain A. (2016). Pediatric Sarcomas. Surg. Clin. N. Am..

[3] Lambin P., Rios-Velazquez E., Leijenaar R., Carvalho S., van Stiphout R.G.P.M., Granton P., Zegers C.M.L., Gillies R., Boellard R., Dekker A. (2012). Radiomics: Extracting More Information from Medical Images Using Advanced Feature Analysis. Eur. J. Cancer.

[4] Crombé A., Fadli D., Italiano A., Saut O., Buy X., Kind M. (2020). Systematic Review of Sarcomas Radiomics Studies: Bridging the Gap between Concepts and Clinical Applications?. Eur. J. Radiol..

[5] Lambin P., Leijenaar R.T.H., Deist T.M., Peerlings J., De Jong E.E.C., Van Timmeren J., Sanduleanu S., Larue R.T.H.M., Even A.J.G., Jochems A. (2017). Radiomics: The Bridge between Medical Imaging and Personalized Medicine. Nat. Rev. Clin. Oncol..

[6] Kocak B., Akinci D’Antonoli T., Mercaldo N., Alberich-Bayarri A., Baessler B., Ambrosini I., Andreychenko A.E., Bakas S., Beets-Tan R.G.H., Bressem K. (2024). METhodological RadiomICs Score (METRICS): A Quality Scoring Tool for Radiomics Research Endorsed by EuSoMII. Insights Imaging.

[7] Zhong J., Hu Y., Si L., Jia G., Xing Y., Zhang H., Yao W. (2021). A Systematic Review of Radiomics in Osteosarcoma: Utilizing Radiomics Quality Score as a Tool Promoting Clinical Translation. Eur. Radiol..

[8] Di Salle G., Tumminello L., Laino M.E., Shalaby S., Aghakhanyan G., Fanni S.C., Febi M., Shortrede J.E., Miccoli M., Faggioni L. (2023). Accuracy of Radiomics in Predicting IDH Mutation Status in Diffuse Gliomas: A Bivariate Meta-Analysis. Radiol. Artif. Intell..

[9] Gitto S., Cuocolo R., Huisman M., Messina C., Albano D., Omoumi P., Kotter E., Maas M., Van Ooijen P., Sconfienza L.M. (2024). CT and MRI Radiomics of Bone and Soft-Tissue Sarcomas: An Updated Systematic Review of Reproducibility and Validation Strategies. Insights Imaging.

[10] Fleiss J.L. (1971). Measuring Nominal Scale Agreement among Many Raters. Psychol. Bull..

[11] Aydos U., Balci E., Ateş S.G., Akdemir Ü.Ö., Karadeniz C., Atay L.Ö. (2023). Quantitative and Visual Analyses of the Effect of Activity Reduction on Image Metrics and Quality in 18F-FDG PET/MRI in Pediatric Oncology. Turk. J. Med. Sci..

[12] Aydos U., Sever T., Vural Ö., Topuz Türkcan B., Okur A., Akdemir Ü.Ö., Poyraz A., Pinarli F.G., Atay L.Ö., Karadeniz C. (2022). Prognostic Value of Fluorodeoxyglucose Positron Emission Tomography Derived Metabolic Parameters and Textural Features in Pediatric Sarcoma. Nucl. Med. Commun..

[13] Bailly C., Leforestier R., Campion L., Thebaud E., Moreau A., Kraeber-Bodere F., Carlier T., Bodet-Milin C. (2017). Prognostic Value of FDG-PET Indices for the Assessment of Histological Response to Neoadjuvant Chemotherapy and Outcome in Pediatric Patients with Ewing Sarcoma and Osteosarcoma. PLoS ONE.

[14] Bouhamama A., Leporq B., Khaled W., Nemeth A., Brahmi M., Dufau J., Marec-Bérard P., Drapé J.-L., Gouin F., Bertrand-Vasseur A. (2022). Prediction of Histologic Neoadjuvant Chemotherapy Response in Osteosarcoma Using Pretherapeutic MRI Radiomics. Radiol. Imaging Cancer.

[15] Cho Y.J., Kim W.S., Choi Y.H., Ha J.Y., Lee S., Park S.J., Cheon J.-E., Kang H.J., Shin H.Y., Kim I.-O. (2019). Computerized Texture Analysis of Pulmonary Nodules in Pediatric Patients with Osteosarcoma: Differentiation of Pulmonary Metastases from Non-Metastatic Nodules. PLoS ONE.

[16] Ding Y., Wang Z., Xu P., Ma Y., Yao W., Li K., Gong Y. (2022). MRI-Based Radiomics in Distinguishing Kaposiform Hemangioendothelioma (KHE) and Fibro-Adipose Vascular Anomaly (FAVA) in Extremities: A Preliminary Retrospective Study. J. Pediatr. Surg..

[17] Giraudo C., Fichera G., Stramare R., Bisogno G., Motta R., Evangelista L., Cecchin D., Zucchetta P. (2022). Radiomic Features as Biomarkers of Soft Tissue Paediatric Sarcomas: Preliminary Results of a PET/MR Study. Radiol. Oncol..

[18] Lin P., Yang P.-F., Chen S., Shao Y.-Y., Xu L., Wu Y., Teng W., Zhou X.-Z., Li B.-H., Luo C. (2020). A Delta-Radiomics Model for Preoperative Evaluation of Neoadjuvant Chemotherapy Response in High-Grade Osteosarcoma. Cancer Imaging.

[19] Sarioglu F.C., Sarioglu O., Guleryuz H., Ozer E., Ince D., Olgun H.N. (2020). MRI-Based Texture Analysis for Differentiating Pediatric Craniofacial Rhabdomyosarcoma from Infantile Hemangioma. Eur. Radiol..

[20] Yang Y., Zhou Y., Zhou C., Ma X. (2022). Novel Computer Aided Diagnostic Models on Multimodality Medical Images to Differentiate Well Differentiated Liposarcomas from Lipomas Approached by Deep Learning Methods. Orphanet J. Rare Dis..

[21] Self C., MacQuarrie K.L., Cost C.R. (2022). Osteosarcoma/Ewing Sarcoma. Pediatr. Rev..

[22] Spadarella G., Stanzione A., Akinci D’Antonoli T., Andreychenko A., Fanni S.C., Ugga L., Kotter E., Cuocolo R. (2022). Systematic Review of the Radiomics Quality Score Applications: An EuSoMII Radiomics Auditing Group Initiative. Eur. Radiol..

[23] Akinci D’Antonoli T., Cavallo A.U., Vernuccio F., Stanzione A., Klontzas M.E., Cannella R., Ugga L., Baran A., Fanni S.C., Petrash E. (2023). Reproducibility of Radiomics Quality Score: An Intra- and Inter-Rater Reliability Study. Eur. Radiol..

